# Prevention and Care Programs Addressing the Growing Prevalence of Diabetes in China

**DOI:** 10.1007/s11892-016-0821-8

**Published:** 2016-10-29

**Authors:** Junmei Yin, Alice P. S. Kong, Juliana C. N. Chan

**Affiliations:** 1 Department of Clinical Nutrition, The First Affiliated Hospital of Sun Yat-Sen University, Guangzhou, China; 2 Asia Diabetes Foundation, Prince of Wales Hospital, Shatin, Hong Kong; 3 Department of Medicine and Therapeutics, The Chinese University of Hong Kong, Prince of Wales Hospital, 9th floor, Lui Che Woo Clinical Science Building, Shatin, Hong Kong; 4 Hong Kong Institute of Diabetes and Obesity, Li Ka Shing Institute of Health Sciences, The Chinese University of Hong Kong, Prince of Wales Hospital, Shatin, Hong Kong

**Keywords:** Diabetes management, Patient empowerment, Peer support, Integrated care

## Abstract

According to a 2010 national survey, 11 % of adults in China have diabetes, affecting 109.6 million individuals. The high prevalence of diabetes has been attributed to the aging of the population, the rapid adoption of energy-dense foods, and a reduction in physical activity. Collectively, these secular changes have created an obesogenic environment that can unmask diabetes in subjects with a genetic predisposition. The growing prevalence of maternal obesity, gestational diabetes, childhood obesity, and early-onset disease can lead to premature morbidity and mortality. Rising to meet these public health challenges, researchers in China have conducted randomized studies to demonstrate the benefits of lifestyle modification in preventing diabetes (the Da Qing Study), as well as that of team-based integrated care, using multiple strategies including peer support and information technology, in order to reduce hospitalizations, cardiovascular-renal complications, and premature deaths. With growing evidence supporting the benefits of these diabetes prevention and management programs, the next challenge is to use policies and systems to scale up the implementation of these programs through raising awareness, building capacity, and providing resources to reduce the human and socioeconomic burden of diabetes.

## Introduction: Diabetes in China

Between 1950 and 1960, China has undergone a period of post-war social hardship followed by a period of famine between 1959 and 1961, and then a long period of social upheaval. In the 1980s, the Chinese Government initiated a series of political and economic reforms that have resulted in phenomenal socioeconomic growth, technological advancement, and cultural changes. The gross domestic product (GDP) increased from 365 billion RMB in 1978 to 56,885 billion RMB in 2013 [1]. With socioeconomic growth, the malnutrition and physical labor experienced in the 1950–1960 was replaced by overconsumption of calories and extensive use of automobiles. These rapid changes in lifestyle are believed to have contributed to the dramatic increase in diabetes prevalence from 0.9 % in 1980 [2] to 11.6 % in 2010 [3•]. Together with the prevalence of gestational diabetes of 8.1 %–10.9 % [4, 5] and childhood obesity of 5.5 % [6], early-onset type 2 diabetes has emerged as a major public health challenge associated with premature morbidity and mortality [7]. This is compounded by an aging society with increasing frailty, which can be amplified by the comorbidities of diabetes in China [8].

## General and Central Obesity

In Asia, the rising prevalence of obesity and metabolic syndrome are major drivers for the increasing incidence [9] and prevalence of diabetes [10–13]. The risk association between body mass index (BMI) and diabetes is particularly robust in individuals below the age of 50 years [14]. Obesity is also an independent risk factor for cardiovascular morbidity and mortality [15]. In a meta-analysis, each 5 kg/m^2^ increase in BMI is associated with 27 % increased risk of coronary heart disease and 18 % increased risk of stroke [16], the latter being the leading cause of death in Asian populations [17]. The rapid lifestyle changes associated with urbanization in China is characterized by high caloric intake and reduced physical activity [18], putting Chinese people at high risk of obesity [19]. The prevalence of adult overweight (BMI ≥25 kg/m^2^) has tripled from 11.7 % in 1991 to 29.2 % in 2009 [20]. Without new public health interventions, by 2030, the prevalence of overweight and obesity are expected to reach 59.7 % and 12.6 %, affecting 669.2 million and 141.2 million people, respectively [20].

Compared with BMI, which is a crude estimate of general obesity, central obesity measured by waist circumference (WC) is a stronger predictor for diabetes in Asia. In Chinese adults aged 18 to 65 years, the mean BMI had increased from 21.5 kg/m^2^ in 1991 to 22.9 kg/m^2^ in 2011, whereas the mean WC had increased from 74.5 cm in 1993 to 80.5 cm in 2011 [20]. For the same BMI, Asian people are more likely to develop diabetes than their Caucasian counterparts, in part due to their tendency to accumulate visceral fat, as reflected by high WC. This ectopic adiposity is associated with abnormal profile of adipokines (eg, low adiponectin) and low grade inflammation (eg, high sensitive C reactive protein), which can cause insulin resistance to unmask diabetes in people with beta cell insufficiency [13, 21]. In Chinese adults, central obesity is a stronger predictor than BMI for coronary heart disease, diabetes, and metabolic syndrome [22–24]. In a 6-year prospective survey, increased WC was associated with 3- to 5-fold increased risk of type 2 diabetes [13]. In a recent study that compared the effect sizes of BMI and WC on health outcomes in Chinese adults, central obesity was associated with increased risk of incident hypertension within normal BMI category. The authors concluded that if only BMI was measured, more than 65 % of people at risk would have been missed [25].

## Diabetes in Young People

In a 2010 Chinese national survey, the prevalence of type 2 diabetes was 4.5 % in people aged 18–29 years and 6.6 % in those aged 30–39 years. In people under the age of 40 years, 40 %–50 % had prediabetes [3•]. In Taiwan and Hong Kong, type 2 diabetes has overtaken type 1 diabetes as the predominant form of diabetes in children [21], similar to that in the West [26]. Early-onset type 2 diabetes substantially increases the risk of premature mortality and multiple morbidities in part due to long disease duration [7, 27] and suboptimal management [28]. In patients with type 2 diabetes diagnosed between the age of 15 and 24 years, the lifetime risk of microalbuminuria approached 100 %, whereas that of blindness was 20 % with an estimated reduced lifespan of 15 years [29].

In China, the rapid nutritional transition in recent decades has major health hazards on both mothers and their offspring. During the famine period of 1959–1961, many women who had suboptimal nutrition during pregnancy gave birth to children who were subsequently exposed to high-fat, calorie-dense diets after birth. In this regard, these offspring had dual exposure to maternal undernutrition followed by childhood and adult overnutrition. Compared with their counterparts born outside this period, offspring of mothers who were pregnant during the famine had 3.9 times increased risk of diabetes or hyperglycemia [30]. This risk was further increased if the offspring had high socioeconomic status later in life or was overweight [30]. These subjects with early-onset type 2 diabetes have high risk of complications due to long disease duration [7, 27]. Yet according to the latest national survey, only 30 % of subjects were diagnosed, and amongst the diagnosed only 25 % were treated, and amongst those treated only 40 % were controlled using a HbA1c of 7 % as recommended target [3•]. Compared with the patients with late-onset diabetes, patients with early-onset diabetes often have suboptimal control of risk factors. These undesirable situations are, in part, due to the silent nature of diabetes and, in part, competing priorities with many young patients being non-adherent and disinclined to have regular medical follow-up visits. Besides, these patients are also less likely to receive intensive treatment despite harboring risk factors and complications resulting in disabilities and premature death [7, 27, 31]. With increasing rates of obesity and physical inactivity among young people across the globe, early-onset type 2 diabetes has become a serious public health issue, calling for urgent actions to detect them early for intervention including regular surveillance and targeted programs.

## Gestational Diabetes Mellitus in China

Gestational diabetes mellitus (GDM) is an important but often overlooked risk condition affecting many women and their offspring. In 2015, more than 20 million live births were affected by diabetes during pregnancy, which accounted for 16.2 % of live births [32]. In China, the Ministry of Health has recommended that providers adopt the diagnostic criteria of GDM based on the International Association of the Diabetes and Pregnancy Study Groups (IADPSG) guidelines since December 2011 [33]. Using these criteria, in 2011, 10.9 % of a cohort of 6201 pregnant Chinese women had GDM [4]. In Tianjin, the fourth largest city of China, which provides universal screening for GDM, the prevalence of GDM has increased from 2.3 % in 1999 to 6.8 % in 2008 and 9.3 % in 2012. However, the magnitude of the problem remains uncertain due to lack of national data [5].

Abnormal glucose tolerance during pregnancy predisposes mother and offspring to metabolic syndrome and type 2 diabetes in later life. On average, women with GDM had more than 7-fold increased risk of developing type 2 diabetes than those with normoglycemia during pregnancy [34]. On the other hand, fetal exposure to intrauterine hyperglycemia is associated with abnormal insulin secretory responses and increased risk of early-onset diabetes, independent of genetic susceptibility and maternal diabetes types [35, 36]. While maternal obesity, GDM, childhood obesity, as well as early-onset diabetes and chronic diseases are global challenges, these secular trends are particularly stark in developing countries like China [37]. In the latest World Health Organization Global Monitoring Framework for Noncommunicable Disease (NCD), a life-course intervention targeting women and children is one of the approaches recommended for the prevention of diabetes and NCD [38].

## Diabetes Prevention and Care Programs in China

Since the late 1980s, researchers in China have conducted a series of trials of interventions, with original designs or adaptations from other international studies, to prevent the onset of diabetes and its complications in order to reduce the disease burden.

### Lifestyle Intervention in High-Risk Subjects

The Da Qing Diabetes Prevention Program was the first randomized study conducted in China to demonstrate the benefits of lifestyle modification on reducing the risk of progression from impaired glucose tolerance (IGT) to diabetes [39]. After 6 years of active intervention, the risk of progression to diabetes was reduced by 51 % compared with the control group irrespective of obesity status at baseline (Table 1). This benefit persisted after 20 years with an annual incidence of diabetes of 7 % in the intervention group versus 11 % in the control group. That said, the 20-year cumulative incidence of diabetes in these subjects with IGT was 80 % in the intervention groups and 93 % in the control group, suggesting some of these subjects might have benefited from earlier intervention, including drugs to halt the progression [40••]. Despite these high cumulative event rates of diabetes, after 23 years, the cumulative incidence of cardiovascular mortality was 11.9 % in the lifestyle intervention group versus 19.6 % in the control group. The respective all-cause mortality rates were 28.1 % vs 38.4 %. These data strongly support the long-term benefits of early intervention in people with IGT, and the need to develop national prevention programs to identify high risk subjects for early intervention [41].

**Table 1:** Effects of lifestyle intervention on prevention of diabetes in three landmark studies [39, 40••, 41–44]

Trials	Subjects with prediabetes (IGT or IFG)		Follow-up period	Incidence of diabetes (intervention versus control)	Extended follow-up period	Incidence of diabetes (intervention versus control)
	Lifestyle intervention	Control		HR(95 % CI)		HR(95 % CI)
Da Qing Study	439	138	6 years	0.49(0.33–0.73)	23 years	0.55(0.40–0.76)
Finnish Diabetes Prevention Study	265	257	3.2 years	0.42(0.30–0.70)	13 years	0.61(0.48–0.79)
Diabetes Prevention Program Study	1079	1082	2.8 years	0.58(0.48–0.66)	15 years	0.73(0.65–0.83)

*CI* confidence interval; *HR* hazard ratio; *IGT/IFG* impaired glucose tolerance/impaired fasting glycemia

The results of the Da Qing Study were highly consistent with the findings of similar trials conducted in Europe and in the United States (Table 1). In the Finnish Diabetes Prevention Study, 522 middle-aged, overweight subjects with IGT were randomized to receive personalized counseling for promoting healthy lifestyle. After a mean follow-up duration of 3.2 years, the risk of developing diabetes was reduced by 58 % in the intervention group [42] with sustained benefits during a median follow-up period of 9 years after the trial completed [43]. In the Diabetes Prevention Program, which involved 3234 American adults with impaired fasting glycemia (IFG) or IGT, the incidence of diabetes was reduced by 58 % with lifestyle intervention and 31 % with metformin after 2.8 years of intervention, compared with the control group, irrespective of gender and race [44]. Similarly, in another study involving Japanese men with IGT, intensive lifestyle modification reduced the risk of diabetes by 67.4 % compared with the control group after 4 years [45].

These encouraging results have provided support to the Chinese national and provincial programs to promote physical activity and healthy eating in adults and children. In 2010, the Ministry of Health launched a comprehensive NCD prevention and control program, including health education and promotion, early detection and treatment, and standardized disease management at the community level. As of 2013, 140 counties in 30 provinces of China had participated in this national program. During this 2-year lifestyle intervention program, there were significant improvements in the participants’ anthropometric measurements and cardiovascular risk profiles [46]. In 2010, the school-based “Take Ten Program” was implemented in 50 primary schools in 6 centers located in the cities of Beijing, Shanghai, Chongqing, as well as provinces of Shandong, Heilongjiang, and Guangdong. This program mandated at least 10-minute moderate to vigorous physical activities every weekday during school attendance. In the pilot study in Beijing involving 500 children, after 1 year of intervention, the proportion of overweight/obese children declined by 0.4 %–5.6 % compared with an increase of 0.6 %–4.5 % in the control group [47, 48]. In 2007, the Department of Education of Shandong Province introduced a new policy of mandatory 1-hour physical activity in school every day. In 2010, 29,030 students aged 10–18 years participated in a survey that divided them into 2 groups (group 1 with physical activity more than 1 h/d and group 2 less than 1 h/). After 1 year of intervention, the prevalence of obesity was 4.99 % in group 1 and 6.62 % in group 2 for boys. The respective figures were 1.57 % and 2.67 % in girls [49]. Given the risk relationship between childhood obesity and diabetes in adulthoods, these school-based programs are likely to promote healthy habits during formative years and is also one of the recommended approaches by the WHO to control childhood obesity [38].

### Patient Empowerment Program

Self-management is a critical component of diabetes care, which includes adjustment of dietary habits, regular physical activity, foot care, self-monitoring of blood glucose and/or urine ketone, taking oral medications and/or injecting insulin, attending regular clinic visits, and managing emotions associated with the demands of living with diabetes [50]. These complex lifestyle changes can be overwhelming for patients if they are not educated, empowered, and engaged regarding the ‘what, why, and how’ of self-management [51]. In the second Diabetes Attitudes, Wishes, and Needs (DAWN2) study conducted in 17 countries, a large proportion of individuals with diabetes expressed lack of confidence to self-manage in terms of changing diets, performing exercise, and taking medications. In China, 60 % of people with diabetes worried about the risk of hypoglycemia, 50 % had diabetes-related distress, but only 40 % had participated in any educational program [52]. In Hong Kong, which is one of the most cosmopolitan cities in southern China, 10 %–50 % of Chinese type 2 diabetic patients attending hospital clinics have depression and/or anxiety, which were associated with increased risk of nonadherence, poor glycemic control, frequent hypoglycemia, hospitalization, and cardiovascular disease [53–57].

Amongst the many challenges of self-management faced by patients with diabetes, lack of knowledge and empowerment are the two most cited barriers [58]. Acquisition of knowledge about diabetes can facilitate self-management, especially in people with low health literacy and limited access to diabetes education. In several systematic reviews, education on self-management plus comprehensive lifestyle interventions improved glycemic and cardiovascular risk factor control [59–61]. Apart from knowledge transfer, patient empowerment is needed to promote one’s ability to think critically and act autonomously. People are empowered when they have sufficient knowledge to make rational choices, adequate resources to implement their decisions, and enough experience to evaluate the effectiveness of their actions [62]. In this connection, patients are in the best position to prioritize their self-management strategies and make adjustments to their lives [63]. Globally, self-management education with patient empowerment strategy is considered to be a key component of diabetes management. Both the American Diabetes Association (ADA) [64] and the United Kingdom National Institute for Health and Clinical Excellence (NICE) Guidelines [65] recommended provision of patient empowerment program upon diagnosis of diabetes

In China, several studies have confirmed the effectiveness of self-management interventions. In a multi-center cross-sectional study of 885 patients with type 2 diabetes conducted in Nanjing, Changsha, Yunnan, and Chongqing, China, patient empowerment was an independent predictor of improved self-care behavior and HbA1c [66]. In Hong Kong, 1141 Chinese patients with type 2 diabetes were enrolled into a large community-based patient empowerment program. After 12 months, the empowerment group achieved greater reduction in HbA1c with 0.307 fewer outpatient clinic visits compared with 0.507 more visits in the matched usual care group [67]. Additionally, the empowerment group had fewer cardiovascular events (1.21 % vs 2.89 %) than the usual care group [68]. The same research group expanded the study and enrolled 27,278 patients with type 2 diabetes without prior history of cardiovascular events into the patient empowerment program. After a median follow-up period of 21.5 months, the empowerment group had a lower rate of all-cause mortality (hazard ratio 0.564, 95 % CI 0.445–0.715) and fewer cardiovascular events (hazard ratio 0.807, 95 % CI 0.696–0.935) compared with patients matched by propensity scores, including age, sex, disease duration, and comorbidities [69].

### Peer Support

Apart from receiving medical treatment from their healthcare providers, patients with diabetes often require ongoing support to translate medical instructions into complex self-care behaviors on a daily basis. By providing informational and emotional support through mutual identification, shared experiences, and increased sense of belonging, peer support has been shown to improve physical and psychological health outcomes. A large number of studies now show that peer support helped patients with diabetes increase physical activity [70, 71], perform self-monitoring of blood glucose [72], adhere to balanced diet [70, 73, 74], and initiate insulin therapy [75]. Peer support has also been shown to improve control of blood glucose [72, 75, 76], blood pressure [77], and blood cholesterol [73], as well as reduce body weight [74] and hypoglycemia [70, 72].

In China, several studies have confirmed the efficacy of peer support in diabetes management. In a pragmatic randomized study involving 3 cities in Anhui Province, within each city, patients in one community were assigned to receive peer support, and in the other community, usual care. The peer supporters were trained by the primary care clinical team and instructed to contact patients within their residential settings. Peer support was well-received, resulting in better diabetes knowledge, self-efficacy, blood pressure, and glucose control [78]. In another randomized trial involving 208 patients with type 2 diabetes receiving community-based insulin therapy in rural China, patients with peer support achieved greater reduction in HbA1c (0.60 % vs 0.32 %), knowledge of insulin usage, and self-management compared with patients with usual care [79]. In a randomized study in Hong Kong, 628 patients with type 2 diabetes who received multi-component integrated care (structured assessment, risk stratification, personalized reporting with decision support, and group empowerment by nurses) were randomized to receive telephone-based peer support for 1 year. Peer support did not further improve control of cardio-metabolic risk factors control but significantly reduced hospitalization [57]. Furthermore, patients who continued to provide peer support to others had better self-care with sustained optimal glycemic control during the 4-year post-trial period than those who decided to quit as peer-supporters after 1 year of the intervention program [80].

People who have to live with lifelong diseases often feel isolated from their network of friends, families, and coworkers, who may not fully understand or empathize with their daily challenges. By sharing their experiences and disclosing their feelings in a warm, sympathetic, and accepting manner, the peer supporters can help their peers to discuss their thoughts and express concerns. With this emotional support, people may change their negative appraisal of life events and gain confidence with reduced negative emotions and increased treatment adherence [81]. Studies have shown that patients with diabetes perceived increased social support after receiving peer support [73, 75]. The potential impacts of these psychosocial–behavioral interventions on health care outcomes and utilizations may be under-recognized. In the aforementioned study of 628 Hong Kong Chinese patients with type 2 diabetes, integrated care and group empowerment improved medication adherence, self-care, and self-efficacy, while reducing negative emotions. Within the peer support group, patients with negative emotions benefited most from peer support with improved psychological health and treatment adherence, which might contribute towards the reduced hospitalization rates [57]. These findings highlight the pluralistic needs of patients with complex disease like diabetes, which require medical care, education, technology, and emotional support. To this end, empowerment and peer support are recommended strategies by the WHO for prevention and control of diabetes [82].

### Care Integration

Variation and fragmentation in healthcare systems are major barriers in effective diabetes management due to phenotypic heterogeneity, complex care protocols, variable standards in clinical expertise, and care provision, compounded by lack of coordination between different levels and settings of care. As a result, diabetic patients often experience difficulty in navigating the health care system with poor care continuity, irrational and inefficient use of resources, unnecessarily expensive interventions, and dissatisfaction of user experiences [83, 84]. The causes of fragmentation vary from one region to another although common causes include institutional segmentation, decentralized administrative entities, disease-specific treatment plans with insufficient coordination, lack of personalized care, and imbalanced resource allocation with priority often given to acute or episodic care [85].

In China, most diabetologists practice in tertiary hospitals whereas primary care physicians, who are more accessible and care for the majority of patients with diabetes, only have a basic knowledge of diabetes. Patients in rural areas have very limited access to comprehensive diabetes assessment, which is mainly provided by diabetologists. In the urban areas, patients often visit multiple hospitals, resulting in both gaps and duplication in service provision. China is a large country with different stages of rural-urban transition and variable insurance coverage in different provinces and cities. Despite their determination to reform the health care system with universal health coverage [86], the Chinese authorities face huge challenges in transforming the long-standing but inefficient, expensive, and fragmented hospital-centric system into one that delivers community-based, high-quality integrated care to address the multiple health care needs associated with urbanization in an aging society [87, 88].

To tackle these problems, experts advocated the use of a multidisciplinary approach to provide comprehensive and coordinated health care to a defined population [89, 90]. From the care provider’s perspective, integrated care may improve clinical effectiveness, responsiveness, acceptability, and efficiency [91, 92]. From the patient’s point of view, integrated care can facilitate timely access to care, prevent service duplication, improve shared decision-making, and promote self-care strategies [93].

**Fig. 1:**
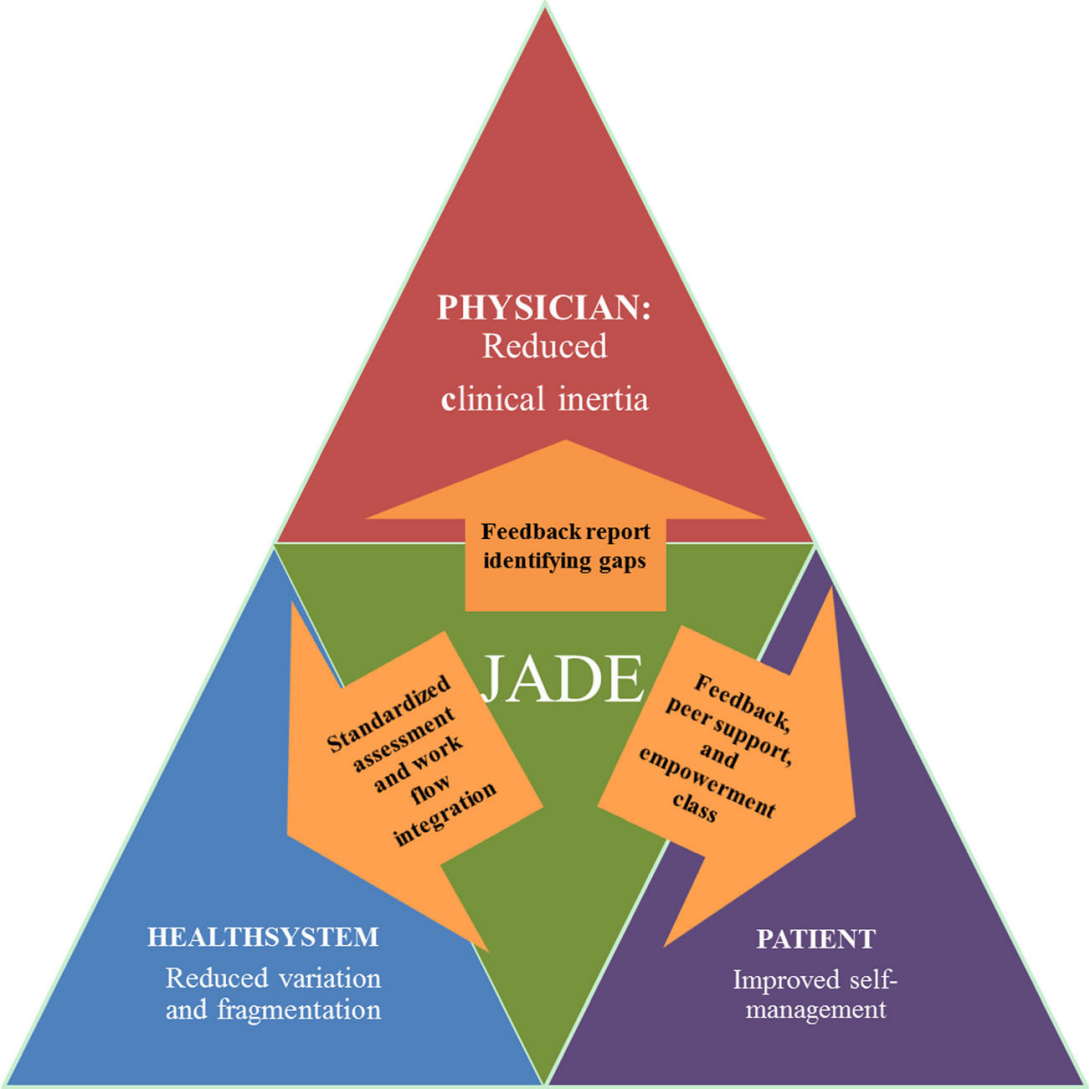
Conceptual framework depicting how integrated care implemented through the Joint Asia Diabetes Evaluation (JADE) Program enabled by a web-based portal might improve outcomes in patients with diabetes

In 2007, using state of the art information technology, researchers in Hong Kong designed the first web-based comprehensive diabetes management program, known as the Joint Asia Diabetes Evaluation (JADE) Program. This program advocates change of workflow and uses trained nurses, under medical supervision, to implement a multi-component quality improvement program in accordance with international practice guidelines [94]. It aims to facilitate delivery of multidisciplinary and coordinated care by specialists, family physicians, nurses, dietitians, podiatrists, community health workers, and trained non-physician personnel, and establish a registry for quality assurance. In order to manage the large amount of clinical information during the clinical course of diabetes, the JADE portal incorporates (1) templates to guide standardized clinical assessment and data capture for risk categorization; (2) validated risk equations to estimate 5-year probabilities of major clinical events; (3) personalized feedback reports with diagrams, secular trends, and bar charts indicating risk profiles, target values, and risk factors control; and (4) individualized decision support for both doctors and patients, triggered by attained target ABC values [glycated hemoglobin (HbA_1c_), blood pressure (BP), and LDL-cholesterol (LDL-C)] and body weight, to empower self-management, reduce clinical inertia, and promote shared decision-making (Fig. 1) [95•].

The JADE Program has provided a care prototype that is highly relevant to this information era where electronic medical systems are increasingly used in both developed and developing countries and areas. These new technologies have enabled healthcare providers to systematically collect, integrate, interpret, and communicate relevant information to educate, engage, and empower patients. It also provides a sustainable platform to conduct multicenter research projects in a real-world setting for evaluating interventions. In a 6-center study in China with each center enrolling 600 patients, each center was given a nurse and clerk to perform comprehensive assessment augmented by the JADE portal with half of them being contacted by their nurse at least twice. After 1 year, there was significant improvement in risk factor control in both groups, with patients receiving additional nurse support having better self care and less likely to default at 1 year [96]. Using the JADE portal to collect and share information, together with patient empowerment and peer support programs, researchers in Hong Kong have demonstrated the benefits of this holistic care model on glycemic control [97], medication adherence, self-care activities, and psychological well-being in people with diabetes [57].

In a 2-year, case-control study involving 160 Chinese patients with diabetic nephropathy, patients who received structured care implemented by a pharmacist-diabetes specialist team with particular emphasis on periodic laboratory assessments, patient adherence, risk factors control, and use of renin angiotensin system (RAS) inhibitors reduced the incidence of end stage renal disease and all-cause death by over 55 % and 78 %, respectively [98]. In another multicenter randomized study involving 205 Chinese patients with type 2 diabetes and renal impairment, more patients in the structured care group attained multiple treatment targets, which were translated to 60 %–70 % risk reduction in premature death and end stage renal disease [99]. In 2009, the Hong Kong Hospital Authority, which manages all public hospitals and clinics in Hong Kong, introduced the Multidisciplinary Risk Assessment and Management Program for Patients with Diabetes Mellitus (RAMP-DM) to evaluate the effectiveness of a multidisciplinary risk-stratification program on metabolic outcomes and cardiovascular events in patients with diabetes within a primary care setting [68]. Compared with a matched cohort adjusted for propensity score, 1248 Chinese type 2 diabetic patients had greater improvements in HbA_1c_ and reduction in cardiovascular risks after receiving 12 months of RAMP-DM. Similarly, 1141 patients with type 2 diabetes received a territory-wide patient-empowerment program (PEP) delivered by non-government organizations [67]. During a 12-month observation period, compared with a matched group under usual care, PEP did not further improve clinical outcomes but reduced health service utilization [67]. These encouraging results have led the Hong Kong Hospital Authority to adopt integrated diabetes care as a corporate strategy. This includes creation of diabetes centers and career track for diabetes specialist nurses for establishing a territory-wide diabetes registry through periodic comprehensive assessment, followed by care triage, patient empowerment, and peer support.

## Conclusions

The causes of the diabetes epidemic in China are complex. These include heterogeneity of genetic, epigenetic, and lifestyle factors against a backdrop of a rapidly changing society with transitions of cultures, behaviors, and environments. In order to meet the medical, social, educational, and emotional needs of these patients, there is a need to establish a holistic care model through multisectorial partnerships for improving quality of care and reducing adverse clinical outcomes. Apart from using information technology to integrate different medical care components with periodic comprehensive assessments, patient empowerment, and peer support are affordable strategies that can be incorporated into such care models. In line with the WHO recommendation, creating a health-enabling environment using public policies, (eg, tobacco control, taxation on sweetened sugar beverages, maternal and child health, environment protection, promoting health literacy, reducing childhood obesity) and strengthening the health care system to provide integrated care, through community empowerment and multisectoral partnerships, hold the keys to success in our common fight to prevent and control diabetes [88].
